# Role of EGF Receptor Regulatory Networks in the Host Response to Viral Infections

**DOI:** 10.3389/fcimb.2021.820355

**Published:** 2022-01-10

**Authors:** Cathleen R. Carlin

**Affiliations:** ^1^ Department of Molecular Biology and Microbiology, School of Medicine, Case Western Reserve University, Cleveland, OH, United States; ^2^ Case Comprehensive Cancer Center, School of Medicine, Case Western Reserve University, Cleveland, OH, United States

**Keywords:** EGF receptor, innate immunity, intracellular trafficking, multivesicular body, viral replication

## Abstract

In this review article, we will first provide a brief overview of EGF receptor (EGFR) structure and function, and its importance as a therapeutic target in epithelial carcinomas. We will then compare what is currently known about canonical EGFR trafficking pathways that are triggered by ligand binding, versus ligand-independent pathways activated by a variety of intrinsic and environmentally induced cellular stresses. Next, we will review the literature regarding the role of EGFR as a host factor with critical roles facilitating viral cell entry and replication. Here we will focus on pathogens exploiting virus-encoded and endogenous EGFR ligands, as well as EGFR-mediated trafficking and signaling pathways that have been co-opted by wild-type viruses and recombinant gene therapy vectors. We will also provide an overview of a recently discovered pathway regulating non-canonical EGFR trafficking and signaling that may be a common feature of viruses like human adenoviruses which signal through p38-mitogen activated protein kinase. We will conclude by discussing the emerging role of EGFR signaling in innate immunity to viral infections, and how viral evasion mechanisms are contributing to our understanding of fundamental EGFR biology.

## Introduction

The purpose of this review is to summarize the multifaceted roles of the epidermal growth factor receptor (EGFR^1^) in the pathogenesis of viral infections. Initially discovered and characterized based on its similarity to the protein tyrosine kinase transforming protein v-ErbB of avian erythroblastosis virus (AEV), early studies were primarily focused on the role of dysregulated EGFR activity as a driver of tumorigenesis. This seminal body of work contributed to the development of tyrosine kinase inhibitors (TKIs), prototypes of targeted therapies that have already led to significant advances in the treatment of numerous human cancers, particularly for patients with non-small-cell lung cancer harboring EGFR activating mutations (Nan et al., 2017). However, the acquisition of resistance to EGFR TKIs is almost inevitable, underscoring the importance of identifying bypass signaling pathways allowing some tumor cells to survive the initial exposure to TKIs and become metastatic (Chang et al., 2016). EGFR-regulated signaling has also emerged as one the most important pathways controlling development and homeostasis particularly in epithelial tissues. It is therefore not surprising that many viruses have developed diverse mechanisms employing EGFR-regulated pathways to invade human cells and transform them into virus-producing factories. Given their clinical importance, it is tempting to consider EGFR-targeted strategies for development of new anti-viral therapies. However, given that these same pathways are fundamentally important for normal physiology, their inhibition will also impair key EGFR functions particularly those involved in repairing cell damage resulting from severe viral infections. Thus, anti-viral drug discovery requires a more nuanced understanding of EGFR-regulated mechanisms contributing to the infection process of multiple biologically diverse animal cell viruses.

## EGFR Signaling and Trafficking Are Inextricably Linked

### Ligand-Induced Regulation of EGFR Activity

EGFR was the first mammalian receptor tyrosine kinase (RTK) discovered, and the founding member of the ErbB RTK family comprising EGFR (or ErbB1), ErbB2, ErbB3, and ErbB4 (Gschwind et al., 2004; Edwin et al., 2006; Linggi and Carpenter, 2006). More than 60 related RTKs, with key roles regulating cellular homeostasis and the development of human diseases when normal physiologic control is disrupted, have now been identified (Hubbard and Till, 2000; Lemmon and Schlessinger, 2010; Esteban-Villarrubia et al., 2020). Most RTKs are single-pass transmembrane proteins with a high affinity extracellular ligand-binding region, a membrane-spanning hydrophobic domain, and an intracellular region (Lemmon and Schlessinger, 2010; Esteban-Villarrubia et al., 2020). In contrast to their highly variable extracellular regions, the cytoplasmic tyrosine kinase catalytic domains of different RTKs are highly conserved, consisting of two lobes with ATP binding and substrate catalysis occurring in a deep cleft between the two lobes (Hubbard and Till, 2000). The cleft also harbors a segment known as the activation loop (A-loop) containing a conserved tyrosine residue. Phosphorylation at the conserved tyrosine residue, which has been shown to help restructure the A-loop into an active conformation, was initially proposed as a unifying mechanism for regulating kinase activation in different RTK families (Hubbard et al., 1998). However, A-loop tyrosine phosphorylation appears to play very different roles depending on the individual RTK (DiNitto et al., 2010). In the case of EGFR, phosphorylation of A-loop tyrosine residue 845 (Tyr845) is not a prerequisite for ligand-induced kinase activation (Gotoh et al., 1992). Early work on EGFR established that ligand binding induced receptor activity by a mechanism involving formation of homodimers or heterodimers with other ErbB family members (Böni-Schnetzler and Pilch, 1987; Yarden and Schlessinger, 1987; Ullrich and Schlessinger, 1990). Studies subsequently showed that ligand binding promoted EGFR kinase activation by an allosteric mechanism, involving the formation of an asymmetric dimer in which one kinase domain drives the other kinase into an active state independently of A-loop Tyr845 phosphorylation (Zhang et al., 2006). Active EGFRs subsequently catalyze autophosphorylation at multiple C-terminal tyrosine residues, creating binding sites for SH2 (Src homology 2) and PTB (phosphotyrosine-binding) domain-containing effector molecules that couple activated receptors to downstream signaling pathways (Yaffe, 2002). Nevertheless, A-loop Tyr845 in the EGFR kinase cleft is a substrate for the non-receptor tyrosine kinase Src (Tice et al., 1999; Sato, 2013). It has been proposed that phospho-Tyr845 and its surrounding amino acid sequence provide a docking site for the SH2 domain of Src enabling formation of EGFR/Src physical complexes. The interaction with Src enhances ligand-induced EGFR signaling in two ways, first by stabilizing the A-loop in a kinase-active configuration (Hubbard et al., 1998); and second by priming EGFR substrates with tandem YY motifs for efficient interaction with the EGFR kinase active site (Begley et al., 2015; Park et al., 2015). Src also plays a critical role in mediating EGFR transactivation downstream of multiple signaling pathways independently of ligand binding (Sato, 2013; Chen et al., 2018).

Ultimately, EGFR signaling is turned off by protein tyrosine phosphatases (PTPs) comprising a structurally diverse family of receptor-like and non-transmembrane enzymes with remarkable specificity for tyrosyl-phosphorylated substrates (Tonks, 2006). The cellular activity of some PTPs, including PTP1B which dephosphorylates multiple RTKs including EGFR, is reversibly inhibited by the reactive oxygen species (ROS) H_2_O_2_ (Xu et al., 2002; Tonks, 2013; Boivin and Tonks, 2015). Since ROS serve as second messengers in multiple signaling pathways, PTP inhibition provides a mechanism for fine-tuning activity of tyrosyl phosphorylated substrates under physiological conditions (Li et al., 2016). PTP1B inactivation *via* ROS may also be an important factor contributing to mis-regulated EGFR signaling elicited by intracellular pathogens that induce oxidative stress to facilitate their own replication and evade immune surveillance (Schwarz, 1996).

### Ligand-Induced Regulation of EGFR Trafficking

**Figure 1:**
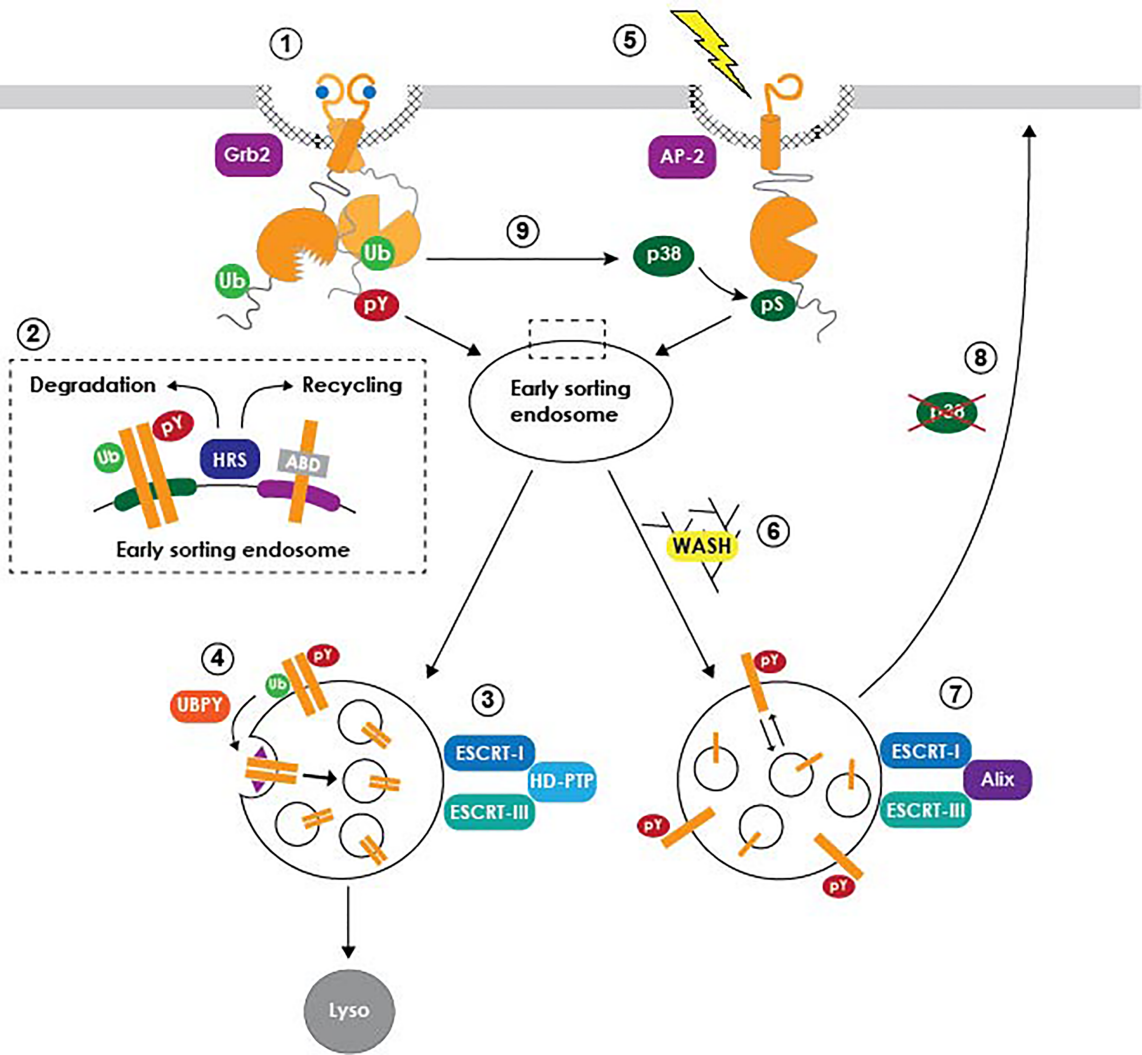
Model summarizing EGFR trafficking pathways. Ligand-activated receptors are internalized from clathrin-coated pits *via* an interaction with Grb2 (1), and subsequently diverted away from a constitutive actin-dependent recycling pathway through their recognition by HRS and other early ubiquitin-binding ESCRT subunits (2). Degradative MVB sorting is regulated by a subset of the ESCRT machinery (3), including accessory proteins such as UBPY that facilitate EGFR transfer from ubiquitin-binding ESCRT machinery to ESCRT-III (4). A putative retention factor (purple triangle) restrains EGFR from being diverted away from nascent ILVs to the ubiquitin-independent recycling pathway. Stress-exposed EGFRs are internalized *via* a p38-MAPK-dependent interaction with the AP-2 clathrin adaptor (5), followed by sustained signaling in non-degradative MVBs *via* a mechanism requiring WASH (6) and Alix (7). Stress-exposed EGFRs subsequently recycle back to plasma membrane following p38-MAPK inactivation (8). Ligand-activated EGFR signaling also regulates endocytic trafficking of unliganded receptor monomers downstream of non-canonical p38-MAPK-dependent phosphorylation. It is not currently known if this pathway involves the same non-degradative MVB sorting mechanism described for stress-exposed EGFRs.

Studies involving EGFR have also been instrumental in unraveling how cell signaling is dynamically regulated by intracellular trafficking (**Figure 1**) (Wiley, 2003; Polo and Di Fiore, 2006; Sorkin and von Zastrow, 2009; Tomas et al., 2014). In the canonical ligand-induced pathway, activated receptors signal on the cell surface relatively briefly before they are endocytosed and subsequently trafficked to lysosomes, where ligand and receptor are both degraded (Gorden et al., 1978; Beguinot et al., 1984). Receptor-mediated endocytosis was initially thought of primarily as a means of signal termination until plasma membrane EGFR levels can be reestablished by *de novo* protein synthesis. However, it is now understood that the EGFR kinase domain retains its activity during its transit in the endocytic system, enabling internalized receptors to continue signaling as long as their activated intracellular domains remain exposed to the cytoplasm (Wiley, 2003; Polo and Di Fiore, 2006; Sorkin and von Zastrow, 2009; Tomas et al., 2014). Studies have also shown that EGFR signaling pathways transmitted at endosomes can be qualitatively different from those originating at the plasma membrane (Wiley, 2003; Polo and Di Fiore, 2006; Sorkin and von Zastrow, 2009; Tomas et al., 2014). Multivesicular bodies (MVBs) are endosome-to-lysosome transport intermediates with a key role in determining the sorting fate of endocytic cargo (Katzmann et al., 2002). MVBs have a characteristic morphology consisting of a cluster of intraluminal vesicles (ILVs) derived by inward invagination from a limiting membrane delineating the interface between the cytoplasm and endosome luminal contents (Dove, 2001). Activated EGFRs are sorted to specialized sub-domains on the MVB limiting membrane that subsequently undergo inward invagination forming ILVs (Miller et al., 1986). In addition to sequestering EGFRs away from cytosolic signaling substrates, ILVs are eventually degraded when MVBs fuse with lysosomes (Futter et al., 1996). MVB sorting is regulated by the concerted action of the ESCRT (Endosomal Sorting Complex Required for Transport) machinery comprising four multi-subunit protein complexes (ESCRT-0, -I, -II, -III) along with a number of accessory proteins (Hurley and Emr, 2006). Degradative sorting is initiated by an interaction between HRS (Hepatocyte growth factor Regulated tyrosine kinase Substrate), a phosphatidylinositol (3)-phosphate binding ESCRT-0 subunit recruited to the surface of early endosomes, and ubiquitin moieties that are attached to the cytosolic tail of activated EGFRs by the E3 ubiquitin ligase c-Cbl (Raiborg and Stenmark, 2002; de Melker et al., 2004). HRS also contributes to EGFR silencing by helping to facilitate formation of inter-organellar membrane contacts that bring tyrosyl phosphorylated EGFR into close proximity with PTP1B anchored in ER (endoplasmic reticulum) membranes (Eden et al., 2010). The ESCRT-I complex acts as a bridge between ESCRT-0 and ESCRT-II, which then contributes to ESCRT-III assembly and subsequent ILV scission from limiting membranes (Bache et al., 2006; Raiborg et al., 2008). However, ligand-activated EGFRs appear to follow an alternative MVB sorting route employing the Bro1 domain-containing cytosolic protein HD-PTP (His domain protein tyrosine phosphatase type N23), acting in place of ESCRT-II to facilitate the transfer of EGFR from ESCRT-0 to ESCRT-III and drive EGFR sorting to ILVs (Ali et al., 2013). ESCRT-II nevertheless has a critical role in EGFR degradation by connecting MVBs to the Rab7 effector** **RILP (Rab7-interacting lysosomal protein), which in turn binds the dynein-dynactin motor complex to coordinate MVB biogenesis with minus end-directed microtubule motility (Progida et al., 2006; Wang and Hong, 2006; Progida et al., 2007).

Paradoxically, HRS is also required for the constitutive recycling of transmembrane proteins including EGFR (**Figure 1**, inset). This trafficking step is regulated by HRS-dependent endosomal recruitment of the actin nucleating factor WASH (Wiscott-Aldrich syndrome protein and SCAR Homologue), which subsequently facilitates recycling *via* direct interactions between endosomal actin and intrinsic actin-binding domains (ABDs) in protein cargo (den Hartigh et al., 1992; MacDonald et al., 2018). Mechanisms driving EGFR trafficking into opposing WASH-mediated receptor recycling and ESCRT-driven degradative pathways remain unclear. For instance, it’s possible that ligand binding inactivates the EGFR ABD, by conformational steric masking or recruitment of an unidentified protein blocking ABD accessibility. Potential ABD blocking proteins include EGFR signaling substrates such as PLCγ (phospholipase C gamma) that are recruited to the Tyr992 autophosphorylation docking site located within the ABD (Tang and Gross, 2003). Alternatively, cooperative interactions between HRS and multiple ubiquitin moieties attached to acutely activated EGFRs may deflect receptors away from a constitutive WASH-mediated recycling pathway. Although still a matter of debate, EGFR deubiquitination by the ESCRT-associated deubiquitinating (DUB) enzyme UBPY (ubiquitin-specific protease 8) appears to facilitate the transfer of activated EGFRs from early ubiquitin-binding ESCRT complexes (ESCRT-0, -I, -II) to ESCRT-III (Mizuno et al., 2005; Row et al., 2006; Alwan and van Leeuwen, 2007). Despite its central role in the degradative sorting pathway, however, UBPY-mediated deubiquitination may make EGFRs vulnerable to re-engagement with an HRS/WASH recycling pathway, unless it is coupled to a mechanism for retaining newly deubiquitinated EGFRs in developing ILVs. A dileucine motif located in the distal EGFR juxtamembrane domain that we have shown to be required for lysosomal degradation represents a candidate MVB retention signal (Kil et al., 1999; Kil and Carlin, 2000; Johnson et al., 2001; Tsacoumangos et al., 2005). Ligand-activated EGFRs with a mutationally inactive dileucine motif are internalized with normal kinetics but then rapidly recycled back to the plasma membrane (Kil et al., 1999; Kil and Carlin, 2000). In addition, this motif behaves as a dominant transferable lysosomal signal for non-RTK membrane cargo (Tsacoumangos et al., 2005). Although the molecular basis of its action remains unknown, structural studies indicate that the EGFR dileucine motif stabilizes an alpha-helix-mediated protein interaction (Tsacoumangos et al., 2005). Interestingly, the juxtamembrane sub-domain harboring the putative EGFR retention motif maps to a region involved in regulating formation of asymmetric kinase dimers during ligand-induced EGFR activation, providing yet another example of the important relationship between EGFR signaling and endocytic trafficking (Red Brewer et al., 2009; Sorkin and von Zastrow, 2009).

EGFR trafficking studies have mostly been carried out in the context of acute stimulation with exogenously applied soluble ligands. However, all seven known EGFR ligands (EGF, TGFA, AREG, EREG, BTC, HBEGF, and EPGN) are synthesized as membrane-anchored precursors (Singh and Coffey, 2014). Except for HBEGF, soluble ligands capable of acting locally on adjacent or nearby cells are released by cleavage in their ectodomains by ADAMs (a disintegrin and metalloproteinase) family members (Singh and Coffey, 2014). Agonist stimulation of G protein-coupled receptors has also been shown to transactivate EGFRs, by increasing metalloproteinase activity and subsequent ligand shedding from the cell surface (Daub et al., 1996). In contrast, surface-exposed HBEGF typically activates EGFR *in trans* at points of cell-cell contact (Singh et al., 2007). TGFA is a high affinity ligand that out-competes EGFR binding to other ligands, and which is likely engaged in homeostatic signaling based on phenotypes in *Tgfa* knock-out mice (Mann et al., 1993). By contrast, the physiological contributions of other ligand-EGFR pairings during normal embryonic development and** **tissue homeostasis/repair are highly context-dependent (Singh et al., 2016). While different ligands all stimulate EGFR internalization, they have very diverse effects on endocytic sorting fates (Roepstorff et al., 2009). Studies defining the canonical trafficking pathway to lysosomes were largely carried out using saturating concentrations of exogenous EGF. However, other ligands such as TGFA and AREG that rapidly dissociate from EGFR in endosomes cause receptor recycling, by a mechanism that in one case (AREG) has been linked to a subset of the ESCRT machinery (ESCRT-I and ESCRT-III) (Schreiber et al., 1986; Baldys and Raymond, 2009). All ligands stimulate EGFR ubiquitination, albeit to different extents, by a mechanism requiring the c-Cbl ubiquitin ligase recruited directly to activated EGFRs at the plasma membrane (de Melker et al., 2001; Roepstorff et al., 2009). However, EGFR ubiquitination is rapidly lost from receptors stimulated with ligands associated with recycling, consistent with the ABD-dependent pathway outcompeting a ubiquitin-driven degradative pathway in HRS-positive endosomes. In addition to UBPY, other DUBs with a potential role in EGFR deubiquitination include the ESCRT-associated enzyme AMSH (associated molecule with the SH3 domain of STAM), and Cezanne-1 which deubiquitinates active EGFRs before they can be efficiently recognized by early ubiquitin binding ESCRT complexes (Meijer et al., 2012; Pareja et al., 2012). In line with a potential role promoting EGFR signaling by opposing receptor degradation, Cezanne-1 overexpression is predictive for aggressive tumor progression in human breast cancer (Pareja et al., 2012). The extent to which various ubiquitin ligases and DUBs contribute to opposing sorting outcomes in ligand-specified EGFR trafficking routes remains to be determined.

### Stress-Induced Regulation of EGFR Trafficking and Signaling

Studies showing that EGFR is also activated by cell stress inducers such as the pro-inflammatory cytokine TNF-α (tumor necrosis factor-alpha), which has a key role in the pathogenesis of many virus-induced diseases, began to appear in the literature nearly two decades ago (Imanishi, 2000; Zwang and Yarden, 2006; Singhirunnusorn et al., 2007; Tan et al., 2016). These ligand-independent EGFR activation pathways are emerging as critical factors in the inevitable development of therapeutic resistance characteristic of most EGFR-driven epithelial carcinomas (Tomas et al., 2014; Tan et al., 2016). Additionally, recent studies have revealed that stress-exposed EGFRs contribute to innate immune responses to viral infections, representing an exciting new area of investigation (Zeng and Carlin, 2019). Stress-exposed receptors are internalized *via* clathrin-mediated endocytosis by a mechanism requiring EGFR phosphorylation on cytosolic Ser/Thr targets of p38-MAPK signaling cascades (Vergarajauregui et al., 2006; Zwang and Yarden, 2006; Grandal et al., 2012). However, ligand- and p38-MAPK-regulated internalization pathways rely on different clathrin adaptors (Grb2 and AP-2 respectively), and p38-MAPK does not induce EGFR ubiquitination (Grandal et al., 2012). Stress-exposed receptors are sorted in a novel population of MVBs that do not fuse with lysosomes, under the control of a subset of ESCRT components including the Bro-domain-containing protein Alix (Tomas et al., 2015). In contrast to EGFR ligands, stress-exposed receptors are activated intracellularly by mechanisms that are not entirely clear. It’s possible EGFR is transactivated by Src following its recruitment to MVB limiting membranes through a known interaction between the Src SH3 (Src homology 3) domain and the proline-rich domain in the Alix C-terminus (Balanis and Carlin, 2017; Hikita et al., 2019). Alternatively, basal EGFR activity may become elevated due to reduced PTP1B activity, either by ROS-induced PTP1B inhibition or failure to form MVB-ER membrane contacts facilitating the interaction between EGFR and PTP1B (Boivin and Tonks, 2015; Tomas et al., 2015). Alix also enables ILV back-fusion with MVB limiting membranes (or retro-fusion), by a process requiring the atypical phospholipid lysobiphosphatidic acid (LBPA) enriched in late endosomes (Matsuo et al., 2004). An Alix/LBPA retro-fusion pathway would provide a potential mechanism for sustaining stress-induced EGFR signaling through continued re-engagement with cytosolic signaling substrates, as well as EGFR recycling back to the plasma membrane following p38-MAPK inactivation. Since the stress-induced pathway sorts non-ubiquitinated EGFR cargo, it seems likely that recycling is mediated through recognition of the EGFR ABD by an HRS/WASH dependent mechanism, although this possibility has not been formally tested (MacDonald et al., 2018). Interestingly, recent studies have shown that the canonical ligand-dependent EGFR signaling pathway induces p38-MAPK-mediated endocytosis of unliganded EGFR monomers, which are largely recycled back to the plasma membrane, as a function of ligand concentration (Tanaka et al., 2018). It is not currently known whether unliganded EGFRs are trafficked through Alix-dependent MVBs and if they can be activated after they have been internalized. Nevertheless, this dual mode of ligand-induced EGFR endocytic trafficking has the potential to cast new light on the relative contributions of MVBs regulating degradative versus stress-induced sorting pathways under the control of HD-PTP and Alix respectively, to EGFR signaling from endosomal platforms.

## Viruses Co-Opting EGFR Ligands

**Figure 2:**
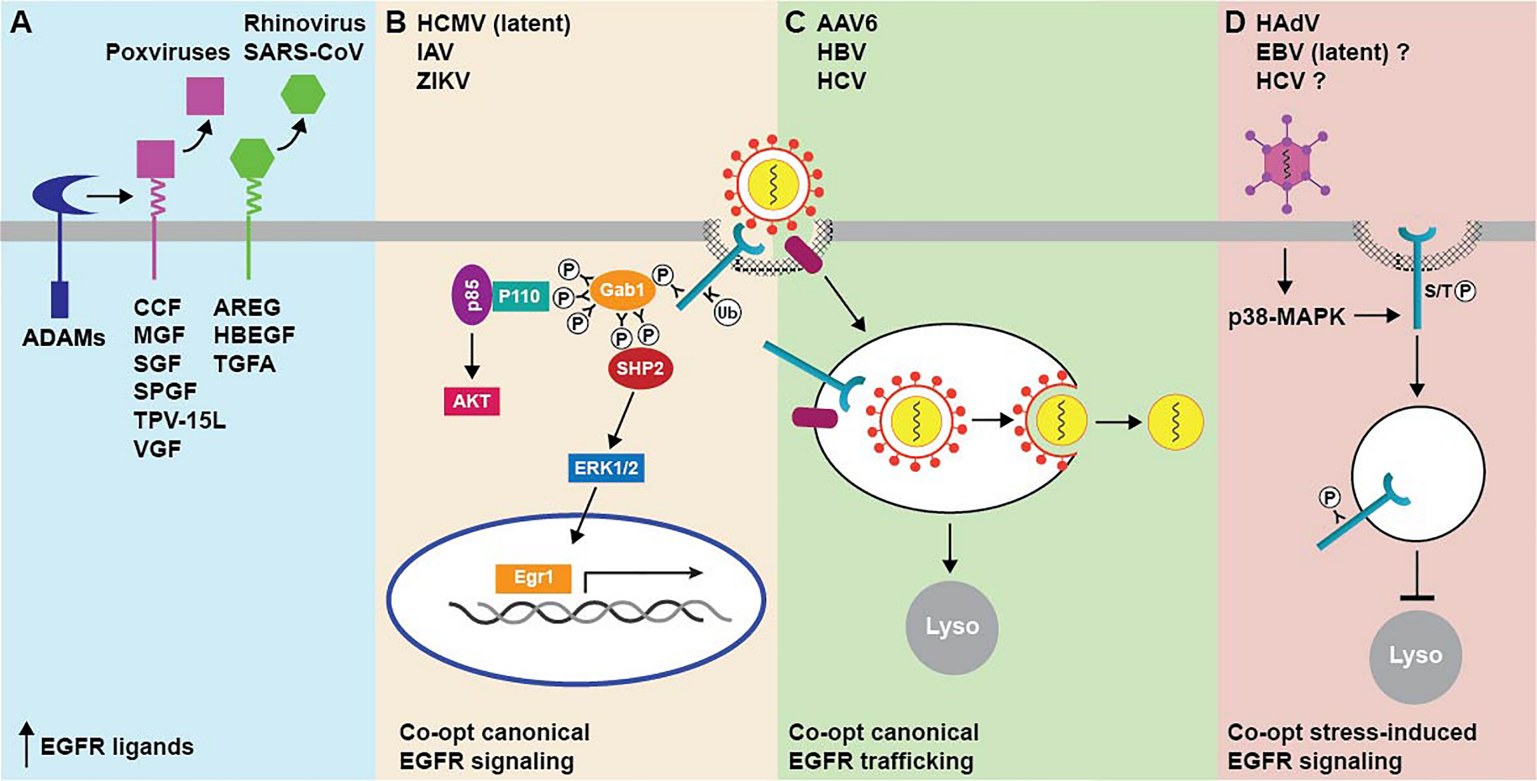
Summary of mechanisms for targeting EGFR by animal cell viruses. **(A)** EGFR activity is regulated by EGF-like growth viruses encoded by multiple poxviruses (magenta), and by up-regulated expression of endogenous EGFR ligands (green) following infection with some respiratory viruses. Mature growth factors released from membrane-bound precursors by host cell ADAMs subsequently act in autocrine and paracrine fashions. **(B)** Virus cell entry requires EGFR-triggered activation of downstream entry relevant signaling pathways, such as PI3K/Akt and ERK-MAPK. Both pathways are linked to EGFR *via* the Gab1 adaptor protein, which activates the p85 catalytic subunit of PI3K by modulating the conformation of the p110 regulatory subunit and also promotes sustained ERK/MAPK signaling downstream of SHP2. **(C)** EGFR facilitates cell entry by linking virus-host cell receptor complexes to endocytic machinery. In the case of enveloped viruses, this may enable trafficking to endocytic compartments with conditions that are favorable for membrane fusion events facilitating nucleocapsid release to the cytosol. **(D)** EGFR does not have a direct role in cell entry but is activated intracellularly in non-degradative endosomes downstream of virus-induced p38-MAPK signaling.

Two general mechanisms for virus-induced EGFR signaling through up-regulated availability of EGFR ligands have been described (**Figure 2A**). One mechanism involves the production of virus-encoded EGF-like ligands, which is characteristic of most members of the poxvirus family of large double-stranded DNA viruses. As a group, poxviruses infect a highly diverse range of vertebrate hosts, causing a broad spectrum of relatively benign to severe skin lesions (Bhanuprakash et al., 2012). In contrast to most other viruses, poxvirus tropism at the cellular level seems to be regulated after the virus has entered the cell and initiated the replication cycle (McFadden, 2005). Poxvirus growth factors have generally been deemed as nonessential for virus replication based on *in vitro* results from viral gene knock-out experiments (Buller et al., 1988a; Buller et al., 1988b, McFadden et al., 1996). Nevertheless, they appear to be important factors contributing to poxvirus virulence, by stimulating mitosis in neighboring quiescent cells effectively priming them for infection once newly synthesized virions have been released from the initial site of infection (Buller et al., 1988a). Vaccinia growth factor (VGF), which is synthesized as a membrane-bound precursor after infection with vaccinia virus, was the first of these products to be identified (Twardzik et al., 1985). Other relatively well described poxvirus EGF-like growth factors include the smallpox growth factor SPGF, Shope fibroma virus growth factor SGF, myxoma virus growth factor MGF, cowpox virus growth factor CGF, and tanapoxvirus growth factor TPV-15L (Upton et al., 1987; Ye et al., 1988; da Fonseca et al., 1999; Kim et al., 2004; Jeng et al., 2013). Although poxvirus growth factors share a resemblance through their EGF-like domains, they are not exchangeable in poxvirus-specific phenotypic assays and have documented differences in ErbB receptor binding specificity (Tzahar et al., 1998). For instance, SFGF is a broad specificity ligand capable of activating all ErbB dimers, VGF preferentially activates EGFR-containing dimers, and MGF and TPV-15L are strictly selective for ErbB2/ErbB3 heterodimers. Although EGFR homodimers are efficiently targeted to lysosomes following EGF engagement, other receptor homo- and heterodimer combinations avoid degradation and instead undergo recycling to the plasma membrane where they may be re-activated (Yarden and Sliwkowski, 2001; Wiley, 2003). These divergent trafficking routes likely account for the fact that most poxvirus growth factors act as potent mitogens, despite having markedly reduced receptor binding affinity compared to endogenous growth factors, since EGFR recycling to plasma membrane facilitates continuous signaling. In addition to a role in tissue tropism, combinatorial poxvirus growth factor/ErbB/effector pathways are likely to be an important factor in determining the innate immune response and resultant pathologies associated with different poxvirus family members.

A second mechanism for regulating EGFR ligand bioavailability is mediated by respiratory viruses that subvert physiological functions of host cell EGFR pro-ligands, which we know have many pivotal roles in epithelial tissue maintenance and repair (Chen et al., 2016). EGFR signaling pathways have been shown to elicit specific protective responses by the lung epithelium, including airway mucin production, IL-8 (interleukin-8) mediated neutrophil recruitment, and airway epithelial repair of injured tissue (Takeyama et al., 1999; Burgel and Nadel, 2004; Nakanaga et al., 2007). Rhinoviruses, which are small non-enveloped positive-strand RNA viruses, appear to co-opt EGFR homeostatic/repair pathways by transcriptionally up-regulating EGFR ligand expression (Bizot et al., 2021). Comprising more than 100 serotypes, rhinoviruses utilize ICAM-1 (intercellular adhesion molecule-1) or LDL receptor for host cell entry (Greve et al., 1989; Suzuki et al., 2001). Typically presenting as a common cold virus associated with only minor self-limiting symptoms, rhinovirus serotypes employing different host cell receptors have also been linked to EGFR-dependent mucus overproduction (Jamieson et al., 2015). Despite an initial benefit in accelerating viral clearance during an acute infection, enhanced mucus production has been shown to be a major factor in the development of virus-induced asthma (Jamieson et al., 2015). Studies have shown that rhinovirus-induced mucin production requires viral replication as well as the pattern recognition receptor TLR3 (Toll-like Receptor 3), which is activated by double-stranded RNA by-products of viral replication (Zhu et al., 2009; Kawasaki and Kawai, 2014). TLR3 typically utilizes the TRIF (TIR-domain-containing adapter-inducing interferon-β) signaling adaptor to connect to downstream signaling pathways (Kawasaki and Kawai, 2014). However, TLR3 appears to transcriptionally up-regulate expression of two EGFR ligands (TGFA and AREG) in cultured and primary lung epithelial cells by a mechanism that is only partially dependent on TRIF expression (Zhu et al., 2009). The secreted EGFR ligands then activate EGFR-MAPK (mitogen-activated protein kinase) signaling leading to enhanced expression of a major airway mucin MUC5AC by a mechanism that is sensitive to ligand-specific neutralizing antibodies, consistent with an autocrine/paracrine mode of action (Zhu et al., 2009). EGFR ligands are also secreted by inflammatory cells, including eosinophils, neutrophils, mast cells, and macrophages, that are recruited to the sites of infection and mechanical trauma (Rappolee et al., 1988; Wong et al., 1990; Calafat et al., 1997; Burgel et al., 2001). Under normal circumstances, these pro-inflammatory responses are downregulated after a successful resolution of wound healing. However, EGFR-regulated responses can become dysregulated and frequently exacerbated by advanced age and underlying lung disease, when the infectious burden becomes overwhelming (Singanayagam et al., 2012). Hyperactive EGFR signaling can then instigate onset and progression of pulmonary fibrosis associated with severe infections caused by respiratory viruses including SARS-associated coronavirus (SARS-CoV) (Jamieson et al., 2015; Venkataraman and Frieman, 2017). Thus, respiratory viruses can induce a spectrum of lung disease ranging from virus-induced asthma to pulmonary fibrosis, by mechanisms that are dependent on endogenous EGFR ligands released from infected epithelial cells, as well as by inflammatory cells elicited by unresolved tissue damage because of viral infection. Both disease-causing mechanisms appear to involve ligands (e.g., AREG and HBEGF) that preferentially bind EGFR homodimers and promote EGFR recycling in favor of degradative sorting, suggesting respiratory viruses co-opt EGFR signaling pathways that are primarily activated at the cell surface by potent EGFR ligands. These studies also support pharmacological disruption of DUBs such as Cezanne-1 that antagonize EGFR degradation as a rationale to potentially target aberrant EGFR signaling induced by respiratory viruses.

## Viruses Utilizing EGFR for Host Cell Entry

EGFR has been identified as an essential host cell co-factor for several viruses (**Figures 2B, C**). In most cases, virus entry requires EGFR-triggered activation of downstream entry relevant signaling pathways. However, EGFR may also have variable roles at different stages of the viral life cycle or act primarily to connect virus to endocytic machinery regulating its uptake. We will discuss influenza virus (IAV), human cytomegalovirus (HCMV), and hepatitis B virus (HBV) respectively, as an example in each of these three categories. EGFR is also an important factor ensuring efficient cell type and tissue-specific infection, as either a native or molecularly targeted host cell co-receptor, in multiple gene therapy applications.

IAV is an enveloped single-stranded RNA virus that is adapted to birds, but which can also sustain bird-to-human and human-to-human transmission and occasionally become pandemic (Krammer et al., 2018). IAVs are taken up by endocytosis following binding of hemagglutinin (HA) on the viral envelop to sialic acids at the host cell surface (Lakadamyali et al., 2004). However, the HA/sialic acid-mediated endocytic route does not occur spontaneously, but requires activation of signaling molecules such as PI3K (phosphoinositide 3-kinase) for efficient uptake (Eierhoff et al., 2010). Recent studies have shown that EGFR is one of several PI3K-inducing RTKs (e.g., c-MET, FGFR) that is activated by IAV attachment (Eierhoff et al., 2010). Moreover, IAV uptake and subsequent infection are sensitive to EGFR activity inhibition. Studies have also revealed that IAV binding leads to clustering of lipid rafts, suggesting that multivalent interactions between HA and sialic acids induce a signaling platform supporting EGFR activation and initiation of downstream signaling cascades (Eierhoff et al., 2010). Consistent with this interpretation, IAV-induced EGFR activation is sensitive to host cell cholesterol depletion but independent of EGFR ligands (Eierhoff et al., 2010). IAV also induces EGFR internalization, suggesting activated receptors engage signals from endosomes as well as from the plasma membrane (Eierhoff et al., 2010). Key EGFR signaling pathways have been proposed as attractive drug targets for blocking IAV replication (O’Hanlon et al., 2019). However, EGFR inhibitors will also impair epithelial tissue regrowth necessary to restore respiratory barrier function after severe infections with IAV and other respiratory viruses associated with hyperactive EGFR signaling, such as respiratory syncytial virus (RSV) and SARS-CoV (Venkataraman et al., 2017; Kalinowski et al., 2018). Recent studies have identified SOCS5 as an important host cell factor capable of limiting IAV infections. SOCS5 is a member of the suppressor of cytokine signaling (SOCS) protein family that primarily act as adaptor proteins to facilitate proteasomal degradation of key signaling substrates. In the case of IAV, SOCS5 has been shown to inhibit EGFR/PI3K activity regulating cell entry. Interestingly, epithelial cells from chronic obstructive pulmonary disease (COPD) patients displaying increased susceptibility to IAV infections have reduced SOCS5 levels, suggesting SOCS5 substrates may represent new anti-viral drug targets (Kedzierski et al., 2017). In addition to IAV, EGFR activation has been shown to be important during early stages of infection with Zika virus (ZIKV), an enveloped RNA virus associated with microcephaly in newborns and other neurological complications, although ERK/MAPK rather than PI3K is the downstream entry relevant signaling pathway (Sabino et al., 2021).

HCMV is a widespread opportunistic herpesvirus with a double-stranded DNA genome that is among the largest among human viruses (Landolfo et al., 2003). While HCMV rarely causes disease in healthy individuals, it can be deadly for the immunocompromised, and is closely associated with the development of atherosclerosis as well as a leading cause of virus-associated birth defects (Griffiths et al., 2015). EGFR signaling inhibits lytic infections and HCMV goes to great lengths to inactivate EGFR, through both transcriptional down-regulation and targeted EGFR degradation (Fairley et al., 2002; Beutler et al., 2003).

In addition to promoting lytic infections in many cell types including fibroblasts and smooth muscle cells, HCMV also establishes latent infections in undifferentiated myeloid lineage cells, such as monocytes and CD34^+^ hematopoietic progenitor cells (Goodrum, 2016). In contrast to its role during lytic infections, HCMV utilizes EGFR signaling for entry and subsequent trafficking of the viral genome to the nucleus in undifferentiated myeloid lineage cells supporting latent infections (Fulkerson et al., 2020). In addition, EGFR is emerging as a key player during HCMV reactivation in latently infected cells. Recent studies have shown that the HCMV-encoded microRNA miR-US5-2 down-regulates the EGFR signaling adaptor protein Gab1, which is the primary mechanism linking EGFR to PI3K signaling in addition to promoting sustained EGFR signaling through MAPK (Cai et al., 2002; Mattoon et al., 2004; Hancock et al., 2020). Gab1 down-regulation serves to restrict activity of the early growth response-1 (Egr1) transcription factor, a downstream target of EGFR/MAPK signaling that regulates expression of the pro-latency HCMV gene product UL138 (Hancock et al., 2020). The hypothesis that miR-US5-2-mediated Gab1 down-regulation has a critical role in switching off the HCMV latency program in favor of a lytic infection is also supported by data showing that pharmacological MAPK and PI3K inhibitors enhance HCMV reactivation in CD34^+^ hematopoietic progenitor cells (Buehler et al., 2019). The ubiquitous herpesvirus Epstein-Barr virus (EBV), which is associated with the development of numerous malignancies, also regulates EGFR during reactivation of latently infected cells but with a different outcome (Chen, 2011). EGFR expression is transcriptionally up-regulated and subsequently activated by the latent membrane protein 1 (LMP1), which is the EBV transforming protein expressed in most EBV-associated malignancies (Kung et al., 2011; Chen et al., 2015b). LMP1-induced cell signaling is quite complex as it involves multiple pathways engaged in significant crosstalk, making it difficult to assign specific functions to EGFR. However, LMP1 acts as a constitutively active TNF-α receptor raising the possibility that stress-activated EGFRs may have an unappreciated role in EBV transformation (Kawanishi, 2000).

HBV is a partially double-stranded DNA enveloped virus representing a global health care problem despite the availability of a vaccine that prevents infections in all age groups for several decades (Liang, 2009). HBV attacks the liver causing acute as well as lifelong chronic infections. Besides hepatitis, infection with HBV can lead to cirrhosis and hepatocellular carcinoma (Liang, 2009). The liver-specific bile acid transporter sodium taurocholate co-transporting polypeptide (NTCP) is the host cell receptor for HBV (Ni et al., 2014). HBV endocytosis is mediated by EGFR, which forms a physical complex with NTCP, by a mechanism requiring EGFR ubiquitination and intrinsic tyrosine kinase activity (Iwamoto et al., 2019). However, rather than requiring downstream signaling molecules such as PI3K or MAPK, HBV relies on the interaction between EGFR and host cell adaptor molecules regulating its intracellular trafficking (e.g., AP2A1, EPS15) for productive infections (Iwamoto et al., 2019; Iwamoto et al., 2020). These results suggest that the EGFR endocytic machinery drives the translocation of NTCP-bound HBV from the cell surface through the endosomal network to late endosomes and lysosomes. Although some studies have suggested that these late endocytic compartments may be the site of membrane fusion facilitating the release of vesicular nucleocapsids into the cytosol, mechanistic details are still being investigated (Watanabe et al., 2007). Linking HBV to a well-characterized EGFR sorting pathway provides a roadmap for identifying roles for additional host cell factors, such as target membrane lipid requirements and endosome-ER membrane contact sites, in the regulation of HBV cell entry. Studies have also revealed a role for EGFR during cell entry of hepatitis C virus (HCV), a small, enveloped, positive-sense single-stranded RNA virus that is a major cause of chronic liver disease worldwide (Houghton, 2019). HCV enters hepatocytes *via* a process requiring the cooperative interaction of several host cell factors including the tetraspanin protein CD81 and the tight junctional protein claudin-1 (Farquhar et al., 2012). RTKs including EGFR and ephrin receptor A2 (EphA2) serve as co-factors for HCV cell entry, by regulating interactions between CD81/claudin-1 co-receptors and the subsequent viral glycoprotein-dependent fusion with host cell membranes (Lupberger et al., 2011; Diao et al., 2012). Although EGFR TKIs have substantial antiviral activity, these inhibitors appear to act primarily by blocking EGFR internalization consistent with EGFR-mediated trafficking as having a critical role in HCV cell entry (Lupberger et al., 2011; Diao et al., 2012). However, HCV cell entry is also associated with transient induction of p38-MAPK signaling raising the possibility that HCV co-opts some of the stress-induced EGFR responses described in next section (Zhao et al., 2007).

Adeno-associated viruses (AAVs) are small single-stranded DNA parvoviruses that serve as the backbone for many gene therapy vectors that are currently in clinical trials (Daya and Berns, 2008; Samulski and Muzyczka, 2014). Initially discovered as a contaminant of human adenovirus (HAdV) preparations, AAV is dependent on co-infection with other viruses to replicate (Naumer et al., 2012; Meier et al., 2020). Recombinant AAVs transduce therapeutic genes, which are inserted in place of viral DNA, into a variety of both dividing and nondividing cells with a relatively low risk of random integration into the host genome (Daya and Berns, 2008; Samulski and Muzyczka, 2014). Added to the fact that AAVs have no known pathogenicity in humans, these characteristics make recombinant AAV vectors ideal for certain gene therapy applications (Verdera et al., 2020). There are several different AAV serotypes, each with the ability to target different cells ranging from kidney cells to neurons in the brain (Wu et al., 2006). Recent studies have shown that EGFR is a specific co-receptor for AAV6, which is commonly used to transduce oncolytic activity into multiple types of tumors including gliomas and lung adenocarcinomas (Huszthy et al., 2005; Gilbert et al., 2008; Weller et al., 2010). EGFR-mediated AAV6 cell entry involves a direct interaction between the AAV6 capsid and EGFR as well as intrinsic EGFR tyrosine kinase activity (Weller et al., 2010). In addition, ectopic EGFR expression is necessary and sufficient for permissive infection of an IL-3-dependent hematopoietic progenitor cell line lacking endogenous EGFR with AAV6, but not with other AAV serotypes (Weller et al., 2010). It is not currently known whether wild-type AAV6 and recombinant AAV6 vectors employ EGFR to specifically engage cellular endocytic machinery, or if they also rely on downstream EGFR signaling pathways, during host cell entry.

There have been several impressive success stories using AAV gene therapy vectors, notably for treatment of inherited retinal diseases (Fuller-Carter et al., 2020). However, success has been somewhat limited because of some major drawbacks of recombinant AAV vectors, including relatively low transduction efficiency and limited genome packaging capacity (Li and Samulski, 2020). In addition, approximately 50% of patients are currently excluded from AAV-based therapies because of pre-existing immunity to viral capsids (Boutin et al., 2010). Self-replicating RNA viruses, particularly alphaviruses, flaviviruses, rhabdoviruses and measles viruses, have emerged as alternative gene therapy vectors to overcome some of these limitations (Khromykh, 2000). Efforts are underway to retarget oncolytic RNA-based virus vectors to tumors cells with elevated expression of EGFR and the constitutively active EGFRvIII mutant associated with poor prognosis in multiple human cancers particularly glioblastoma multiforme (GBM) (Gan et al., 2013). EGFR retargeting strategies include incorporating EGFR adaptors, such as a single chain EGFR antibody or an EGFR ligand, into the viral capsid (Paraskevakou et al., 2007; Nakano et al., 2011). Although rational design parameters are still being established, EGFR adaptors have the potential to modify function of specific cells within mixed cell populations.

## Viruses Co-Opting Stress-Induced EGFR Signaling

**Figure 3:**
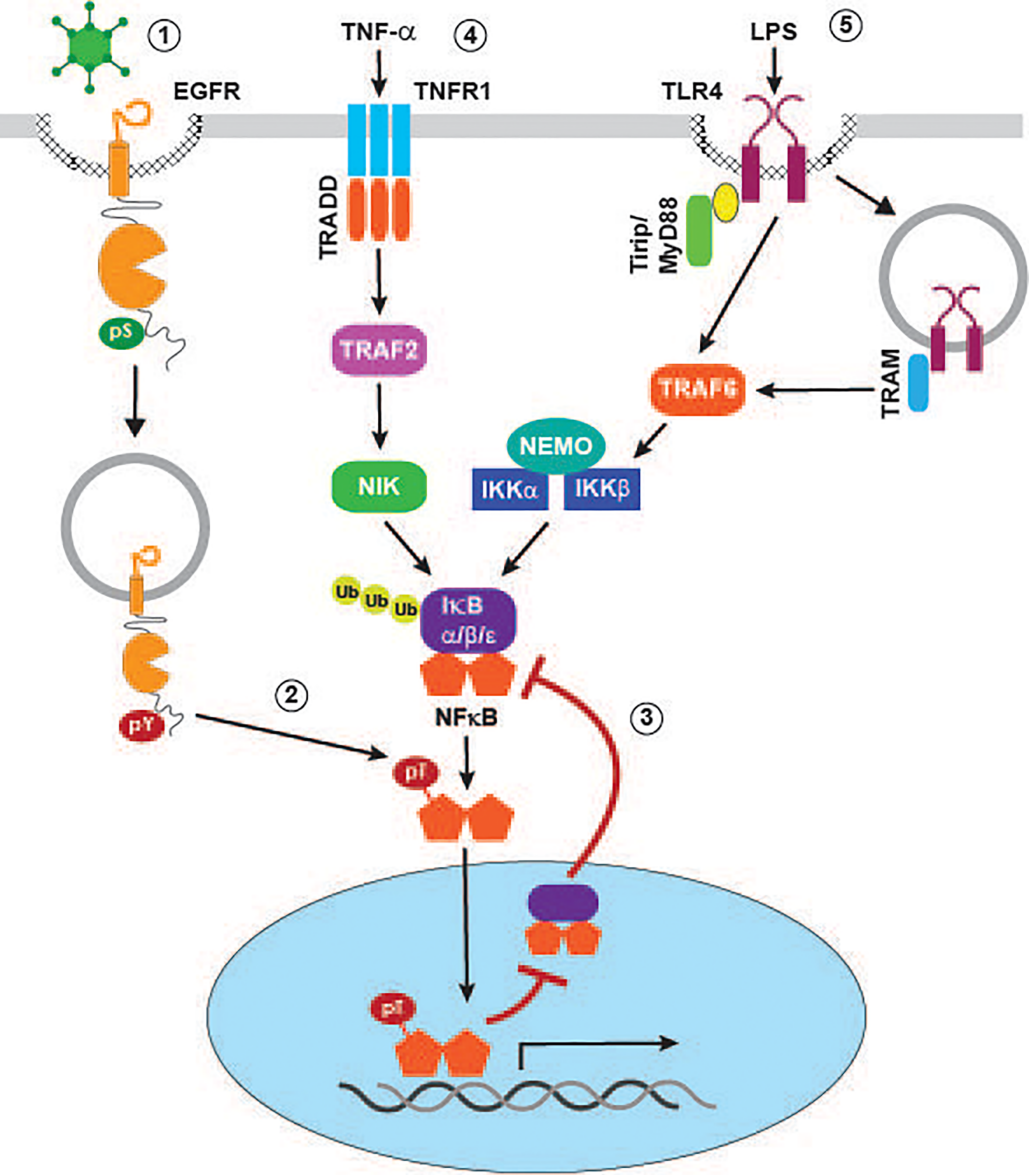
Signaling crosstalk regulating pro-inflammatory NF-κB responses in virally infected cells. p38-MAPK activating viruses such as HAdV induce EGFR internalization to a non-degradative MVB subpopulation where receptors are subsequently activated independent of ligand binding (1). Stress-activated EGFRs mediate phosphorylation of the p65 NF-κB subunit at a Thr-Pro site (2), leading to inhibition of feedback control that is normally facilitated by formation of NF-κB/IκBα complexes in the nucleus (3). TNF-α (4) and LPS (5) activate canonical NF-κB signaling pathways by mechanisms described in the text.

Numerous enveloped and non-enveloped viruses have been shown to activate one or more members of the MAPK family. In some instances, as is the case with herpes simplex virus type 1 (HSV-1), activation of c-Jun N-terminal kinase (JNK) requires viral protein synthesis (McLean and Bachenheimer, 1999). However, transient MAPK activity also occurs as a direct consequence of viral binding to host cell receptors independent of viral protein synthesis (**Figure 2D**). This mechanism is illustrated by HAdV type C serotypes associated with relatively mild self-limiting upper respiratory tract infections (e.g., HAdV-C2 and HAdV-C5), which are known to trigger transient activation of p38-MAPK and its downstream target MAPKAP kinase 2 (MK2) during early stages of cell penetration (Horwitz, 1995; Suomalainen et al., 2001). The p38-MAPK/MK2 pathway was originally characterized for its ability to promote nuclear targeting of incoming viral particles on minus-end directed microtubules (Suomalainen et al., 2001). Our recent studies have provided evidence supporting an additional role for the HAdV/p38-MAPK/MK2 signaling pathway, by mediating ligand-independent EGFR activation in non-immune airway epithelial cells. Furthermore, HAdV-induced EGFR activity is associated with an early wave of NF-κB (nuclear factor kappa-light-chain-enhancer of activated B cells) signaling (Zeng and Carlin, 2019). Although NF-κB signaling is generally thought of as pro-inflammatory, it can also be protective by shielding epithelial cells from inflammation caused by innate immune cells recruited to the surface of infected cells (Pasparakis, 2009). NF-κB is normally sequestered in the cytoplasm *via* a noncovalent interaction with inhibitory IκB (nuclear factor of kappa light polypeptide gene enhancer in B-cells inhibitor) proteins that mask NF-κB nuclear localization signals (Ghosh and Karin, 2002; Li and Verma, 2002). NF-κB signaling is activated by a variety of stimuli that eventually activate IKKs (IκB kinases), which can then phosphorylate inhibitory IκB proteins leading to their degradation and subsequent release of NF-κB subunits for nuclear translocation (Ghosh and Karin, 2002; Li and Verma, 2002). However, rather than activating the canonical pathway, our studies have revealed that HAdV-induced EGFR signaling interferes with a major negative feedback mechanism involving NF-κB-regulated *de novo* synthesis of IκBα, which can remove NF-κB from cognate promoters and shuttle it back to the cytoplasm (**Figure 3**) (Ghosh and Karin, 2002; Li and Verma, 2002). The HAdV-induced EGFR signaling pathway causes phosphorylation of the p65 subunit of NF-κB at a Thr254-Pro motif before the onset of early viral protein (e.g., E1A) expression (Zeng and Carlin, 2019). Prior to our studies, other investigators had shown that phospho-Thr254-Pro was a binding site for the prolyl isomerase Pin1, and that it antagonized the interaction of p65 with IκBα (Ryo et al., 2003). Consistent with those findings, HAdV-induced EGFR signaling was associated with a significant increase in nuclear accumulation and protein stability of p65 accompanied by up-regulated NF-κB transcriptional activity, using synthesis of the pro-inflammatory cytokine IL-8 as a readout, compared to mock-infected epithelial cells (Zeng and Carlin, 2019). NF-κB transcriptional activity was subsequently attenuated following expression of an HAdV-C2/5 encoded early gene product called E3-RIDα, a small endosome-localized membrane protein which we had previously identified based on its ability to divert EGFRs away from the plasma membrane to lysosomes (Hoffman et al., 1992a; Hoffman et al., 1992b; Hoffman and Carlin, 1994; Crooks et al., 2000; Zeng and Carlin, 2019). Several key findings suggest E3-RIDα redirects MVBs regulating the trafficking and signaling of stress-exposed EGFRs to a degradative pathway. First, similar to other p38-MAPK activating cell stresses, the E3-RIDα regulated EGFR sorting pathway is mediated by Alix acting in place of ESCRT-II (Zeng and Carlin, 2019). Second, the E3-RIDα-regulated pathway is dependent on the previously described EGFR dileucine-based retention signal required for efficient sorting in the degradative MVB pathway (Tsacoumangos et al., 2005). Third, E3-RIDα forms a molecular complex with the Rab7 effector RILP, which is normally recruited to degradative MVBs through its interaction with ESCRT-II (Shah et al., 2007). Fourth, the RILP domain involved in targeting the** **dynein-dynactin motor complex regulating the biogenesis of Rab7-containing organelles was required for E3-RIDα-induced EGFR down-regulation in infected cells (Progida et al., 2006; Wang and Hong, 2006; Shah et al., 2007). Finally, ectopic E3-RIDα expression is sufficient to reconstitute degradative EGFR sorting in Rab7-depleted cells (Shah et al., 2007; Zeng and Carlin, 2019). We also showed that HAdV serotypes associated with severe and occasionally fatal infections (e.g., HAdV-E4 and HAdV-B7) provoke a sustained pro-inflammatory EGFR/NF-κB/IL-8 response compared to HAdV-C2/5 (Horwitz, 1995; Zeng and Carlin, 2019). Strikingly, these pathogenic HAdV serotypes encode E3-RIDα proteins that have divergent cytosolic tail sequences and do not promote EGFR degradation (Zeng and Carlin, 2019). Overall, our published studies support a working model that the E3-RIDα encoded by HAdV serotypes that are generally asymptomatic restore negative feedback control to NF-κB signaling, by antagonizing a stress-induced EGFR pathway associated with p65 accumulation in the nucleus and enhanced NF-κB activity. We believe this is important for striking a balance between an initially beneficial wave of NF-κB signaling and immune-mediated tissue injury (Ginsberg et al., 1989; Pasparakis, 2009).

## EGFR and Innate Immunity to Viral Infections

NF-κB signaling, which is a hallmark of most viral infections, is involved in immune cell development and function, as well as modulation of immune responses of infected epithelial cells (Santoro et al., 2003). NF-κB target gene products with a central role in innate and adaptive immune responses include a wide spectrum of cytokines and chemokines that mediate the exacerbated inflammation triggered by the host in response to viral infections, receptors required for neutrophil adhesion and transmigration across blood vessel walls, members of the major histocompatibility complex (MHC), and proteins involved in antigen presentation (Pahl, 1999). NF-κB also regulates expression of IκBα, which mediates the previously described negative feedback mechanism limiting NF-κB transcriptional activity (Ghosh and Karin, 2002; Li and Verma, 2002). Several mechanisms have now been identified linking EGFR signaling to canonical pathways of NF-κB activation, mostly in the context of cancer cells (Shostak and Chariot, 2015) . For instance, the EGFR/PLCγ pathway activates IKKs by a mechanism involving an interaction between PKCε (protein kinase C epsilon) and the IKKα/IKKβ scaffolding protein NEMO (Yang et al., 2012). This pathway was discovered in cancer cells overexpressing the NF-κB target pyruvate kinase M2 (PKM2), a glycolytic enzyme which allows survival of cancer cells under low oxygen conditions (Mazurek et al., 2005; Yang et al., 2012). However, it is currently unknown whether any of these EGFR/canonical NF-κB pathways are activated by viruses that up-regulate expression of virus-encoded and endogenous EGFR ligands, or which activate EGFR co-receptors during cell entry.

We have already described our recent studies uncovering a novel pathway linking stress-activated EGFRs to the NF-κB pathway by interfering with IκB negative feedback control of NF-κB transcriptional activity (Zeng and Carlin, 2019). In addition to providing a mechanism for increasing basal NF-κB signaling, the stress-induced EGFR pathway may amplify inflammatory responses induced by stimuli that activate a canonical NF-κB activation pathway during viral infections (**Figure 3**). For instance, the pro-inflammatory cytokine TNF-α, which is released from immune cells recruited to sites of infection by TNF-α-converting enzyme (TACE), has a major role in viral pathogenesis and disease (Imanishi, 2000). This has led to significant efforts aimed at identifying host cell and virus-induced factors capable of modulating canonical TNF-α signaling pathways downstream of cognate TNFR1 and TNFR2 receptors (Herbein and O'Brien, 2000). The TNF-α signal transduction pathway mediating NF-κB activation involves the TNFR1 adaptor TRAF (TNF receptor-associated factor) and NIK (TNF receptor-associated factor and NF-κB inducing kinase), which in turn drives phosphorylation-dependent degradation of IκB inhibitory proteins. In addition to crosstalk with the virus-activated EGFR pathway, it is likely that TNF-α signaling establishes a positive autoregulatory loop by directly inducing stress-activated EGFR signaling (Zwang and Yarden, 2006; Singhirunnusorn et al., 2007). Lipopolysaccharide (LPS) binding triggers two TLR4 pathways, one involving the MyD88 adaptor protein recruited to TLR4 at the plasma membrane, and a second engaging the adaptor protein TRIF after TLR4 endocytosis. Both pathways have been linked to NF-κB activation by mechanisms converging on the downstream signaling adaptor TRAF6 (Lu et al., 2008). Interestingly, we have shown that ectopic expression the HAdV-encoded RIDα protein, which downregulates stress-activated EGFRs, attenuates TNF-α/NF-κB signaling in uninfected respiratory epithelial cells, as well as LPS-induced TLR4/NF-κB signaling in HAdV-infected cells (Cianciola et al., 2017; Zeng and Carlin, 2019). These finding suggests that the HAdV-RIDα protein may have co-opted a physiological mechanism allowing for dynamic interconversion between MVB subpopulations regulating EGFR activity by determining receptor sorting fates.

EGFR is also involved in innate immunity pathways regulated by TLR3, which is localized to endosomes where it recognizes double-stranded RNA (dsRNA) replication intemediates produced during viral infections. In contrast to other TLRs, TLR3 requires phosphorylation of two specific tyrosine residues in its cytoplasmic domain to recruit the adaptor protein TRIF linking it to antiviral innate immune signaling (Sarkar et al., 2007). Studies have shown that EGFR docks to the linker region domain in the TLR3 cytosolic tail that is exposed by dsRNA binding (Yamashita et al., 2012). EGFR subsequently recruits Src and the two protein tyrosine kinases act coordinately to phosphorylate TLR3 tyrosine residues allowing TRIF recruitment. This working model assumes EGFRs located in endosomal membranes under basal conditions are sufficient to facilitate TRLR3/TRIF signaling in response to dsRNA binding. However, it is also possible this innate immune signaling pathway is further enhanced by viruses that co-opt EGFR endocytic machinery to facilitate host cell entry. It also remains to be seen if EGFR links dsRNA-activated TLR3 to PI3K signaling, which is known to be required for the full activation of TLR3-induced transcriptional responses (Sarkar et al., 2004; Sarkar et al., 2007).

In contrast to its roles in activating innate immune signaling, EGFR has also been shown to counteract these responses in some instances. For example, EGFR signaling limits antiviral activity associated with DDX60 (DExD/H-Box Helicase 60), an RNA helicase that recognizes short viral dsRNA in the cytosol during a viral infection. dsRNA-bound DDX60 subsequently promotes signaling by the cytosolic pattern recognition receptor RIG-I (retinoic acid-inducible gene I) responsible for the type-1 interferon anti-viral response (Rehwinkel and Gack, 2020). Recent studies have shown that viruses which activate EGFR during cell entry, such as IAV and HCV, attenuate DDX60/RIG-1 signaling by promoting EGFR-dependent DDX60 tyrosine phosphorylation which effectively blocks DDX60 function (Oshiumi et al., 2015). A second example of EGFR-mediated immune suppression involves human papillomaviruses (HPVs) that infect keratinocytes. In this case, HPV upregulates EGFR activity to drive expression of IRFD1 (interferon-related developmental regulator 1). IRFD1 subsequently impairs the acetylation of the NF-κB RelA subunit, which is required for full activation of the pro-inflammatory cytokine response and immune cell attraction to HPV-infected keratinocytes (Tummers et al., 2015). Interestingly, HPV induces two phases of EGFR signaling during cell entry, a transient activation shortly after cell binding that likely induces a stress-induced EGFR pathway followed by a second wave of signaling which is thought to drive HPV endocytosis (Bannach et al., 2020). It remains to be seen if either of these EGFR pathways are specifically linked to up-regulated IRFD1 expression.

## Concluding Remarks

It is now abundantly clear that multiple viruses engage EGFR-regulated pathways to facilitate their replication and modulate host defense responses. Among mechanisms discussed in this review are *de novo* production of virus-encoded EGF-like growth factors and transcriptional up-regulation of endogenous EGFR ligands. Viruses have also been shown to co-opt the EGFR endocytic apparatus and activate cell entry relevant EGFR signaling pathways to gain access to the host cell cytosol. In addition, viruses exploit the heterogenous nature of EGFR signaling networks by engaging different ligand/receptor dimer combinations to control virus tissue tropism and EGFR signaling potency. EGFR is also being exploited for the rational design of gene therapy vectors targeting cells with pathological EGFR expression. In the interest of fostering antiviral drug discovery, it is important to compare how different viruses take over EGFR trafficking and signaling pathways for their own benefit with an eye towards identifying common themes.

Historically virologists have focused on the canonical pathway regulating the trafficking of ligand-activated EGFRs from the plasma membrane to lysosomes as a roadmap for discovering the role of EGFR during virus infections. However, stress-induced EGFR trafficking pathways that have recently emerged as important factors controlling host-virus interactions also merit consideration. For instance, EGFR-mediated uptake of HBV likely involves a stress-regulated pathway because of its dependence on the clathrin adaptor AP2A1, which is known to be a specific requirement for clathrin-mediated internalization of stress-exposed EGFRs (Grandal et al., 2012; Iwamoto et al., 2019; Iwamoto et al., 2020). Besides a reliance on a particular clathrin adaptor, another useful marker for determining whether receptors are being trafficked in degradative or cell stress-associated pathways is the identity of the Bro domain-containing ESCRT accessory protein regulating virus-induced EGFR sorting in MVBs (requiring HD-PTP and Alix respectively). However, more work is needed to molecularly dissect these virus-induced pathways, to clarify how they are regulated and the interplay between different EGFR trafficking pathways, and whether they can be targeted to develop new anti‐viral therapies. For instance, drugs capable of blocking EGFR trafficking in the canonical ligand-induced pathway may be beneficial for attacking viruses that need to reach late endosomes and lysosomes, where the conditions are sufficient to trigger a viral fusion protein which in some cases involves proteolytic priming. On the other hand, forcing non-degradative MVBs into a degradative pathway may be a useful drug development strategy targeting viruses that activate EGFR signaling pathways supporting their replication by inducing cell stress.

Some investigators have proposed the repurposing of FDA-approved EGFR TKIs as antivirals (Hondermarck et al., 2020). However, this strategy has some major drawbacks if EGFR signaling pathways engaged in repairing virus-damaged tissue are also inhibited. In addition, except for viruses that co-opt EGFR ligands, exactly how EGFR receptors become activated following exposure to viruses remains an open question in most infectious settings. For instance, it is not clear whether TKIs targeting ligand-activated EGFRs that are currently in clinical trials will be as effective in inhibiting stress-exposed EGFRs that are transactivated intracellularly following virus-induced internalization. In addition, despite decades of work supporting roles for ROS in viral pathogenesis, it remains unknown whether PTP1B inhibition is a viable mechanism supporting ligand-independent EGFR activation following exposure to stress-inducing viruses. The list of viruses for which ROS are thought to play a role in their pathogenesis include IAV, human immunodeficiency virus (HIV), and HBV which has been shown to elicit an approximately 10,000-fold increase in intracellular ROS in chronically infected hepatocytes (Chen and Siddiqui, 2007; Cho et al., 2011; Ivanov et al., 2016; Xu et al., 2017). In addition, the effect of viral infection on formation of productive interactions between internalized receptors and PTP1B regulating EGFR dephosphorylation at endosome-ER membrane contacts sites remains unknown.

It is also emerging that EGFRs represent a signaling hub for transducing host cell pathways with special relevance for viral replication. Studies carried out so far have examined these pathways following infection with wild-type viruses. However, these same pathways are likely to be induced by oncolytic recombinant gene therapy vectors targeting EGFR-overexpressing tumor, where they may have unanticipated consequences. For instance, multiple viruses appear to converge on the multi-site docking protein Gab1, which integrates PI3K and MAPK signaling downstream of EGFR and other receptors, including the CD14 pattern recognition receptor regulating production of proinflammatory cytokines during TLR4 signaling in macrophages (**Figure 2B**) (Cai et al., 2002; Mattoon et al., 2004; Li et al., 2015; Hancock et al., 2020). However, as is often the case, individual viruses co-opt EGFR-Gab1 functions to achieve different outcomes. For instance, we’ve already discussed that HCMV down-regulates Gab1 to facilitate its reactivation from latency (Hancock et al., 2020). In contrast, polyomavirus activates the Gab1/PI3K pathway to promote endothelial cell transformation and coxsackievirus B3 co-opts Gab1/MAPK signaling to enhance its infectivity (Ong et al., 2001; Deng et al., 2015). Consistent with an important role in the host innate immune response to viral infections, Gab1 is required for vesicular stomatitis virus (VSV) infection-induced IFN-alpha/beta production (Li et al., 2015). In addition, Gab1 may be activated at the plasma membrane as well as in endosomes, suggesting it regulates EGFR signaling in multiple trafficking pathways (Rodrigues et al., 2000; Kostenko et al., 2006). Coupled with the fact that Gab1 is frequently overexpressed in human cancers, it’s central role in multiple signaling pathways has made Gab1 an attractive candidate for therapeutic drug discovery. However, it remains to be determined whether Gab1 inhibitors have antiviral activity (Chen et al., 2015a).

Recent evidence from our laboratory has demonstrated that stress-activated EGFRs instigate a non-canonical pro-inflammatory NF-κB pathway contributing to the early innate immune response of epithelial cells following their exposure to HAdVs (Zeng and Carlin, 2019). However, the molecular details of the downstream EGFR signaling cascade mediating this response remains unknown. Another important question is whether stress-activated EGFR is a signaling nexus integrating multiple innate immune responses to viral infections. Ligands for NKG2D, a prototypic innate immunity receptor constitutively expressed on human NK cells and T lymphocytes that promotes killing by NK cells once activated, represent an interesting case-in-point (Zingoni et al., 2018). NKG2D ligand expression is negligible in normal tissues but increased in stress and disease conditions for reasons that until recently were incompletely understood. Stress-inducing UVB irradiation has now been shown to up-regulate epithelial surface expression of NKG2D ligands by a mechanism that is attributable to ligand-independent EGFR activation (Vantourout et al., 2014). These findings support a possible role for virus-induced EGFR signaling in NK cell-mediated killing of virally infected cells (Prager and Watzl, 2019).

One of the more fascinating challenges for virologists is understanding what host-pathogen interactions can tell us about normal cellular physiology, and the interplay between EGFR and animal cell viruses is no exception. For instance, it is currently unclear whether EGFRs that are sorted by distinctive endocytic machinery elicit the same signals, albeit with distinct rates of turnover, or if they are qualitatively different. However, evidence emerging from the study of animal cell viruses suggests that pathways regulating innate immune responses, which may be associated with viral pathogenesis if left unchecked, are weighted towards stress-exposed EGFRs signaling from non-degradative MVBs (Zeng and Carlin, 2019). Another open question in EGFR biology asks how cells integrate signals generated by a plethora of factors enabling EGFR activity *via* different mechanisms to effectively maintain homeostasis. Presumably, EGFR activity is normally controlled by the balanced trafficking of receptors and signaling molecules through different MVB subpopulations. The existence of stringent feed-back control mechanisms regulating communication between these compartments would theoretically allow cells to make dynamic adjustments to EGFR signaling in response to a change in the cellular environment. Under normal circumstances, feedback control would reassert homeostatic EGFR signaling after a successful resolution of the initial triggering event. However, EGFR-regulated responses could become dysregulated and exacerbated when mechanisms responsible for maintaining a proper balance of signaling elicited from different endocytic compartments become compromised, representing a conceptually new approach to development of EGFR-targeted antivirals.

## Author Contributions

The author confirms being the sole contributor of this work and has approved it for publication.

## Funding

This work was supported by Public Health Service RO1 Grants GM138696, NS104103, and DK125115.

## Conflict of Interest

The author declares that the research was conducted in the absence of any commercial or financial relationships that could be construed as a potential conflict of interest.

## Publisher’s Note

All claims expressed in this article are solely those of the authors and do not necessarily represent those of their affiliated organizations, or those of the publisher, the editors and the reviewers. Any product that may be evaluated in this article, or claim that may be made by its manufacturer, is not guaranteed or endorsed by the publisher.

## Glossary

**Table:**

AAV	adeno-associated virus
ABD	actin-binding domain
ADAM	a disintegrin and metalloproteinase
AEV	avian erythroblastosis virus
AMSH	associated molecule with the SH3 domain of STAM
CGF	cowpox virus growth factor
COPD	chronic obstructive pulmonary disease
DDX60	DExD/H-Box Helicase 60
DUB	deubiquitinating
EB	Epstein-Barr virus
EGFR	epidermal growth factor receptor
EGR1	early growth response-1
ER	endoplasmic reticulum
ESCRT	endosomal sorting complex required for transport
GBM	glioblastoma multiforme
HA	hemagglutinin
HBV	hepatitis virus B
HCMV	human cytomegalovirus
HCV	hepatitis virus C
HD-PTP	His domain protein tyrosine phosphatase type N23
HIV	human immunodeficiency virus
HRS	hepatocyte growth factor regulated tyrosine kinase substrate
HPV	human papillomavirus
HSV-1	herpes simplex virus type 1
IAV	influenza virus
ICAM-1	intercellular adhesion molecule-1
IκB	nuclear factor of kappa light polypeptide gene enhancer in B-cells inhibitor
IKK	IκB kinase
IL-8	interleukin-8
ILV	intraluminal vesicle
IRFD1	interferon-related developmental regulator 1
JNK	c-Jun N-terminal kinase
LBPA	lysobisphosphatidic acid
LMP1	latent membrane protein 1
LPS	lipopolysaccharide
MAPK	mitogen-activated protein kinase
MGF	myxoma virus growth factor
MHC	major histocompatibility complex
MK2	MAPKAP kinase 2
MVB	multivesicular body
NF-κB	nuclear factor kappa-light-chain-enhancer of activated B cells
NTCP	sodium taurocholate co-transporting polypeptide
PI3K	phosphoinositide 3 kinase
PKCε	protein kinase C epsilon
PLCγ	phospholipase C gamma
PTB	phosphotyrosine-binding
PTP	protein tyrosine phosphatase
RIG-1	retinoic acid-inducible gene I
RILP	Rab7 interacting protein
ROS	reactive oxygen species
RSV	respiratory syncytial virus
RTK	receptor tyrosine kinase
SARS-CoV	SARS-associated coronavirus
SGF	Shope fibroma virus growth factor
SH2/3	Src-homology 2/3
SOCS	suppressor of cytokine signaling
SPGF	smallpox growth factor
TKI	tyrosine kinase inhibitor
TLR	Toll-like receptor
TNF-α	tumor necrosis factor-alpha
TPV-15L	tanapoxvirus growth factor
TRIF	TIR-domain-containing adapter-inducing interferon-β
UBPY	ubiquitin-specific protease 8
VGF	Vaccinia growth factor
VSV	vesicular stomatitis virus
WASH	Wiscott-Aldrich syndrome protein and SCAR homologue
ZIKV	Zika virus
